# Role of cancer ratio and other new parameters in the differential diagnosis of malignant pleural effusion

**DOI:** 10.6061/clinics/2021/e2515

**Published:** 2021-04-16

**Authors:** Zenghua Ren, Ling Xu

**Affiliations:** Department of Respiratory Medicine, Shanghai Jiao Tong University Affiliated Sixth People's Hospital, Shanghai, China.

**Keywords:** Malignant Pleural Effusion, Cancer Ratio, Adenosine Deaminase, Lactate Dehydrogenase, Differential Diagnosis

## Abstract

**OBJECTIVES:**

We compared the diagnostic potential of cancer ratio (CR, serum lactate dehydrogenase [LDH]/pleural fluid adenosine deaminase [pfADA]), cancer ratio plus (CR plus, cancer ratio/pleural lymphocyte percentage), and age/pfADA ratio with pfADA in malignant pleural effusion.

**METHODS:**

Data from 100 patients with malignant pleural effusion (MPE) and 119 patients with tuberculous pleural effusion (TPE) were retrospectively collected. PfADA, age/pfADA ratio, CR, and CR plus were compared between patients with MPE and those with TPE in two age groups (≤50 and >50 years). The best cut-off value was determined, and the diagnostic performance was evaluated according to the receiver operating characteristic curve.

**RESULTS:**

PfADA was statistically significantly lower while age/pfADA ratio, CR, and CR plus were significantly higher in the MPE group than in the TPE group in both age groups (*p*<0.05). For patients aged ≤50 years, the differential diagnostic value of pfADA for MPE was better than those of age/pfADA ratio, CR, and CR plus. At a cut-off value of 13.0 U/L, the sensitivity, specificity, and accuracy were 88.9%, 100.0%, and 98.9%, respectively. For patients aged >50 years, the diagnostic performance of CR plus was superior to those of pfADA, age/pfADA ratio, and CR. At a cut-off value of 22.6, the sensitivity, specificity, and accuracy of CR plus for the diagnosis of MPE were 86.8%, 84.6%, and 86.2%, respectively.

**CONCLUSIONS:**

The best parameter for diagnosing MPE was different for patients aged ≤50 years and >50 years. For patients aged >50 years, CR plus was a good parameter for the differential diagnosis of MPE. For patients aged ≤50 years, pfADA was better.

## INTRODUCTION

Malignant pleural effusion (MPE) is caused by malignant tumors originating in the pleura or metastasis of malignant tumors from other locations to the pleura. Once diagnosed with MPE, the median survival is only 3-12 months (1). Pleural fluid cytological or pleural pathological examination is helpful for the diagnosis of MPE, but the positive rate of MPE is approximately 60% (2). Although thoracoscopic or closed pleural biopsy improves diagnostic sensitivity, it is not only traumatic with the risk of complications but also requires high cost and technical requirements. New diagnostic techniques are limited by factors such as diagnostic efficiency, technical requirements, and cost. For example, Tian et al. (3) studied the diagnostic value of survivin, a tumor-regulated inhibitor protein, for the diagnosis of MPE. The results showed that although survivin was elevated in MPE and the diagnostic specificity was acceptable (88.89%), the sensitivity was only 57.50%. Therefore, finding an economical and practical parameter for the differential diagnosis of MPE has important clinical application value. In the study by Verma et al. (4), the serum lactate dehydrogenase (sLDH)/pleural fluid adenosine deaminase (pfADA) ratio had a high sensitivity and specificity for diagnosing MPE and it was called “cancer ratio (CR).” Subsequently, Verma et al. (5) conducted a prospective study and found that CR maintained and “CR plus” (ratio of CR and pleural fluid lymphocyte count) improved the specificity of CR in identifying MPE. Similar to MPE, tuberculous pleural effusion (TPE) is another type of lymphocyte-predominant exudative pleural effusion. It is often necessary to distinguish MPE from TPE in clinical settings, especially when pleural fluid cytological and microbiological results are negative. Although the above studies included TPE as the main type of pleural effusion distinguished from MPE, they did not compare the diagnostic value of pfADA, a specific biomarker of TPE, with CR and CR plus. Therefore, further studies are required to determine if pfADA, CR, CR plus, or any other parameter is more valuable for the differential diagnosis of MPE and TPE. Based on the above, the objective of this study was to compare the diagnostic performance of parameters such as pfADA, age/pfADA, CR, and CR plus, and to identify the best parameter to identify MPE.

## MATERIALS AND METHODS

This study was approved by the Ethics Committee of Shanghai Jiao Tong University Affiliated Sixth People's Hospital. Data from patients with MPE who underwent thoracentesis between January 2003 and May 2020 were retrospectively collected. Patients with TPE were included in the control group.

MPE was diagnosed if pleural effusion was exudative and met one of the following criteria (6): (1) malignant cells were found in lung tissues and (2) malignant cells were found in pleural fluid or pleural tissues.

TPE diagnosis was confirmed when pleural effusion was exudative and met one of the following conditions (6-8): (1) positive smear for acid-fast bacilli in pleural fluid/sputum/bronchial aspirate/bronchoscopic brushing specimen; (2) positive culture or positive polymerase chain reaction for *Mycobacterium tuberculosis* in pleural fluid/sputum/bronchial aspirate; (3) epithelioid caseous granuloma or positive acid-fast staining in pleural or lung tissue; (4) moderately or strongly positive 5U tuberculin skin test, positive T-cell spot test, or positive *M. tuberculosis* antibody test and a clinical response to anti-tuberculosis treatment; or (5) typical symptoms of tuberculosis with no evidence of additional respiratory diseases and a marked response to anti-tuberculosis treatment. A clinical response to anti-tuberculosis treatment refers to the relief of symptoms, remission, or elimination of pleural effusion in patients who have been followed up for at least 12 months after receiving anti-tuberculosis treatment.

The exclusion criteria were as follows: (1) patients with transudative pleural effusion; (2) patients without pfADA, sLDH, or pleural fluid lymphocyte count results; and (3) patients who were unable to provide information during follow-up visits in the TPE group.

We collected parameters including patient's sex and age, sLDH, and laboratory test results of pleural fluid (total and differential cell count, and ADA). Three new parameters were calculated. They were age/pfADA ratio, CR (sLDH/pfADA ratio), and CR plus (sLDH/pfADA/pleural fluid L% ratio).

ADA activity was measured by enzymatic colorimetry (ADA kit, Junshi Biotechnology Co., Ltd., Shanghai, China). LDH levels were measured using the lactic acid substrate method (LDH assay kit, DiaSys Diagnostic Systems Shanghai Co., Ltd, Germany). Total white blood cells in the pleural fluid were measured using a Sysmex XN-3000 Automatic Blood Analyzer. Differential cell counts in pleural fluid were counted manually.

### Statistical Analyses

Statistical analysis was performed using SPSS version 20.0 software (SPSS Inc., Chicago, IL, USA) and MedCalc version 9.2.1.0 software (MedCalc Software, Broekstraat 52, B-9030 Mariakerke, Belgium). Data are presented as medians (25^th^, 75^th^ percentile) for continuous variables or frequencies (proportions) for categorical variables. The Kruskal-Wallis H test was used for comparison among three groups, Mann-Whitney U test was used for comparison between the two groups, and chi-square test was used for comparison between qualitative variables. Statistical significance was set at *p*<0.05. The correlations between sex, age, and the four parameters were analyzed using Spearman correlation. The best cut-off values and diagnostic performance were evaluated based on the receiver operating characteristic (ROC) curve. Differences in the areas under the ROC curve (AUCs) were compared using MedCalc 9.2.1.0.

## RESULTS

### Patient characteristics

A total of 219 patients with exudative pleural effusion were analyzed: 100 (45.7%) had MPE and 119 (54.3%) had TPE. Among those with MPE, the etiology of malignancy was as follows: lung cancer (82 patients), breast cancer (5 patients), thyroid cancer (2 patients), pleural mesothelioma (3 patients), lymphoma (1 patient), gastric cancer (1 patient), liver cancer (1 patient), pleural bidirectional differentiation malignant tumor (epithelioid hemangioendothelioma) (1 patient), papillary carcinoma of the nasal cavity (1 patient), endometrial carcinoma (1 patient), and adenocarcinoma with unknown primary site (2 patients).

Males were predominant in both groups. The median age of the MPE group was higher than that of the TPE group. There were significant differences in age and sex between the groups of patients with MPE and TPE (Table 1).

### Correlation analysis between sex/age and pfADA, age/pfADA ratio, CR, and CR plus

To clarify the influence of sex and age on the four parameters, we performed a Spearman correlation analysis. The results showed that none of the parameters in the total sample were sex-related, although all of them were age-related (Table 2).

### Comparison of pfADA, age/pfADA ratio, CR, and CR plus in patients with MPE and TPE

Considering that age influenced the four parameters, we divided the patients into three age groups (≤30, 31-50, and >50 years) and compared the differences in these four parameters among the three age groups. The results showed that there was no difference in pfADA, CR, and CR plus between the ≤30 year- and 31-50 year-age groups (Table 3). Therefore, we divided patients into two age subgroups (≤50 and >50 years) to compare the differences in the four parameters between the MPE and TPE groups (Table 4). We found that the level of pfADA in the MPE group was significantly lower than that in the TPE group, while the age/pfADA ratio, CR, and CR plus were significantly higher than those in the MPE group (*p*<0.05).

### Diagnostic performance of pfADA, age/pfADA ratio, CR, and CR plus for MPE

The diagnostic performance of pfADA, age/pfADA ratio, CR, and CR plus for MPE in the ≤50 year- and >50 year-age groups is shown in Table 5. There was no significant difference in the AUC between the four parameters (Table 6). Considering both sensitivity and specificity, the best parameters for the diagnosis of MPE were different in the ≤50 year- and >50 year-age groups. For patients aged ≤50 years, pfADA and age/pfADA had the same diagnostic performance, both of which were superior to CR and CR plus. However, in terms of convenience, the pfADA was better. At a cut-off value of 13.0 U/L, the sensitivity, specificity, and accuracy were 88.9%, 100.0%, and 98.9%, respectively. For patients >50 years, the diagnostic performance of CR plus was superior to that of the other three parameters in terms of both sensitivity and specificity. At a cut-off value of 22.6, the sensitivity, specificity, and accuracy were 86.8%, 84.6%, and 86.2%, respectively.

## DISCUSSION

In this study, we compared the values of pfADA, age/pfADA, CR, and CR plus in the differential diagnosis of MPE and TPE. We found that the age of the patients was significantly different between the two groups, and these four parameters were all age-related. To eliminate the effect of age on the results, we divided the study population into ≤50 year- and >50 year-age groups for subgroup analysis. The results showed that the best parameter for diagnosing MPE was different in the ≤50 year- and >50 year-age groups. In the >50 year-age group, our results were consistent with those of Verma et al. (5). In other words, CR plus showed the best diagnostic performance for MPE. However, in the ≤50 year-age group, the diagnostic performance of CR and CR plus was not better than that of pfADA.

The discussion about each parameter was as follows:

1. pfADA: ADA is a key enzyme in purine metabolism that catalyzes the conversion of adenosine to inosine (9). It is secreted by monocytes, lymphocytes, neutrophils, and red blood cells. Inflammation occurs in the pleura of patients with TPE; then, the body's cellular immunity is enhanced and T lymphocytes are activated. Therefore, the ADA level of patients with TPE increased (10). Unlike TPE, the immune function of patients with cancer is suppressed, resulting in a decrease in the activity of ADA in lymphocytes (11). A large number of studies have shown that pfADA is elevated in patients with TPE and has a good diagnostic value for TPE (12-14). Zhang et al. (13) studied the value of cytokines, tumor markers, and biochemical markers in pleural fluid in the differential diagnosis of MPE and TPE. The results showed that the AUC of pfADA was larger than those of most markers, including tumor necrosis factor alpha, interleukin-6, cytokeratin-19 fragment, carcinoembryonic antigen, and C-reactive protein, and was second only to that of interferon-γ (IFN-γ). However, compared with pfADA, the measurement of IFN-γ is expensive, which limits its use in many developing countries. Therefore, pfADA is still a clinically useful marker for identifying MPE and TPE. In our study, for patients aged ≤50 years, pfADA and age/ADA had higher diagnostic specificity and accuracy than CR and CR plus, and the sensitivity did not decrease. Compared with pfADA, the introduction of age did not increase diagnostic performance. Therefore, from the perspective of clinical practice, pfADA is better. For patients aged >50 years, although the specificity of pfADA was the best, the sensitivity and accuracy were the lowest among the four parameters. A lower sensitivity may lead to missed diagnoses. Therefore, considering both sensitivity and specificity, the diagnostic performance of pfADA was not good for patients aged >50 years.
2. Age/pfADA ratio: In our study, age was negatively correlated with pfADA (r=-0.521, *p*=0.000), which was consistent with the results of Abrao et al. (8). Patients with MPE were older than patients with TPE, while pfADA levels were lower. Therefore, the age/pfADA ratio was expected to expand the difference between the two groups and to improve diagnostic performance. In the study by Korczynski et al. (15), the sensitivity and specificity of the age/pfADA ratio for diagnosing MPE were 93.2% and 71.2%, respectively. In our study, age/pfADA did not improve diagnostic performance in patients aged ≤50 years when compared with pfADA. For patients aged >50 years, the sensitivity, specificity, and accuracy of age/pfADA for diagnosing MPE were 81.3%, 89.7%, and 83.8%, respectively. Compared with pfADA, the specificity decreased by 2.6%, although the sensitivity increased by 6.6%.
3. CR (sLDH/pfADA ratio): LDH mediates the production of ATP in the anaerobic glycolytic pathway. Tumor cells preferentially produce energy via this pathway. Therefore, LDH levels in patients with MPE are often elevated (4). At the same time, pfADA levels in patients with MPE were lower than those in patients with TPE. Therefore, the ratio of sLDH/pfADA is expected to improve the diagnostic performance. Verma et al. (4) reported that the sLDH/pfADA ratio was significantly increased in patients with MPE. When the ratio was ≥20.0, exudative pleural effusion could be highly predicted to be malignant, with high sensitivity and specificity (98.0% and 94.0%, respectively). Therefore, the authors named this ratio: CR. Korczynski et al. (15) also conducted a similar study and found that when the ratio was >16.4, the sensitivity for diagnosing MPE was 94.6%, the specificity was 68.2%, the platelet-lymphocyte ratio was 2.97, and the neutrophil-lymphocyte ratio was 0.08. The sensitivity of sLDH/pfADA in both studies was similar, although the specificity in the study by Korczynski et al. was much lower than that in the study by Verma et al. In addition, these studies did not perform a subgroup analysis according to age. Our study showed that for patients aged ≤50 years, the best cut-off value was 12.9, with a sensitivity of 88.9%, a specificity of 88.8%, and an accuracy of 88.8%. Although the sensitivity did not decrease, the specificity and accuracy of CR for the diagnosis of MPE were much lower than those of pfADA. For patients aged >50 years, the best cut-off value was 19.2, with a sensitivity of 81.3%, a specificity of 87.2%, and an accuracy of 83.1%. The sensitivity was the same as that of age/pfADA, although the specificity slightly decreased.
4. CR plus (sLDH/pfADA/pleural fluid L% ratio): Verma et al. (5) conducted a prospective cohort study to assess the value of sLDH/pfADA/pleural fluid lymphocyte count in the differential diagnosis of lymphocyte-predominant MPE and lymphocyte-predominant TPE. It was found that pleural effusion could be highly predicted as MPE when the ratio was >30, with high sensitivity (97.0%) and specificity (94.0%). Our study showed that CR plus was significantly higher in the MPE group than in the TPE group. For patients aged ≤50 years, the sensitivity of CR plus for diagnosis of MPE was 88.9%, the specificity was 92.5%, and the accuracy was 92.1% at a cut-off value of 25.0. Compared with pfADA, the specificity and accuracy were reduced, and the sensitivity did not increase. For patients aged >50 years, the best cut-off value of CR plus for diagnosis of MPE was 22.6, with a sensitivity of 86.8%, a specificity of 84.6%, and an accuracy of 86.2%. Compared with pfADA, age/pfADA, and CR, the sensitivity and accuracy were increased, and the specificity was not significantly decreased, which helped reduce missed diagnosis and had higher clinical practical value. Compared with the study by Verma et al. (5), our study population was not confined to lymphocyte-predominant MPE and TPE, but also included neutrophil-predominant TPE and MPE. Therefore, our results may be more applicable than those reported by Verma et al. In addition, we performed subgroup analysis by age, which could rule out the influence of age on the diagnostic efficiency of this parameter; therefore, our results were more reliable.

In summary, considering both sensitivity and specificity, pfADA was the best parameter for the differential diagnosis of MPE in patients aged ≤50 years, while CR plus was the best parameter for patients aged >50 years. Compared with pfADA, age/pfADA, and CR, CR plus significantly improved sensitivity, which helped to reduce missed diagnoses. The parameters used in our study were derived from routine biochemical tests, which are simple, cheap, and time-saving. Our findings can guide physicians in differentiating between patients with MPE and those with TPE, and initiate treatment as soon as possible.

Our study had some limitations. First, this was a single-center retrospective study with unavoidable selection bias. Second, our study population included only patients with MPE and TPE and did not enroll patients with exudative pleural effusion from other causes (such as connective tissue disease) to verify these results. Third, most patients with MPE had lung cancer, and only one case was lymphoma. Because lymphoma-associated MPE can also lead to higher pfADA, more patients with lymphoma are needed for further study.

## AUTHOR CONTRIBUTIONS

All authors contributed to the conception and design of the study. Material preparation, data collection, and analysis were performed by Xu L and Ren Z. The first draft of the manuscript was written by Ren Z and Xu L commented on the previous versions of the manuscript. All of the authors read and approved the final version of the manuscript.

## Figures and Tables

**Table 1:** General characteristics of patients with MPE and TPE.

		MPE (n=100)	TPE (n=119)	*p* value
Sex	Male	52 (52.0)	82 (68.9)	0.011
	Female	48 (48.0)	37 (31.1)	
Age (years)		72.0 (61.0, 77.8)	31.0 (24.0, 59.0)	0.000

Data in the table are expressed as frequencies (%) and medians (interquartile ranges).

TPE: tuberculous pleural effusion; MPE: malignant pleural effusion.

**Table 2:** Correlation analysis between sex/age and pfADA, age/pfADA ratio, CR, and CR plus in all patients.

	Sex		Age	
	r value	*p* value	r value	*p* value
pfADA	-0.056	0.411	-0.521	0.000
Age/pfADA	0.031	0.653	0.732	0.000
CR	-0.004	0.951	0.480	0.000
CR plus	0.000	0.998	0.482	0.000

pfADA: pleural fluid adenosine deaminase; CR: sLDH/pfADA ratio, and CR plus (sLDH/pfADA / pleural fluid L% ratio).

**Table 3:** Comparison of pfADA, age/pfADA, CR, and CR plus in patients aged ≤30, 31-50 and >50 years.

	Age ≤30 years (A, n=59)	Age 31-50 years (B, n=30)	Age >50 years (C, n=130)	*p* value		
				A *vs* B	A *vs* C	B *vs* C
PfADA	28.0 (24.0, 33.0)	24.0 (12.8, 42.8)	9.0 (4.8, 18.0)	0.411	0.000	0.000
Age/pfADA	0.9 (0.7, 1.1)	1.5 (0.8, 3.5)	8.3 (4.0, 15.1)	0.015	0.000	0.000
CR	7.4 (5.1, 9.4)	8.5 (4.7, 21.3)	27.2 (10.4, 53.3)	0.410	0.000	0.000
CR plus	0.1 (0.1, 0.1)	0.1 (0.1, 0.4)	0.4 (0.2, 0.8)	0.108	0.000	0.003

pfADA: pleural fluid adenosine deaminase; CR: sLDH/pfADA ratio; and CR plus: sLDH/pfADA/pleural fluid L% ratio.

**Table 4:** Comparison of pfADA, age/pfADA ratio, CR, and CR plus in patients with MPE and TPE.

	Age ≤50 years			Age >50 years		
	MPE (n=9)	TPE (n=80)	*p* value	MPE (n=91)	TPE (n=39)	*p* value
pfADA (U/L)	8.0 (5.0, 12.5)	28.0 (23.3, 36.0)	0.000	6.0 (4.0, 10.0)	19.0 (13.0, 26.0)	0.000
age/pfADA	5.9 (3.7, 8.9)	0.9 (0.7, 1.1)	0.000	11.5 (7.6, 19.3)	3.7 (2.8, 4.5)	0.000
CR	35.5 (20.3, 55.4)	7.3 (4.8, 9.3)	0.000	38.0 (20.6, 62.0)	9.9 (7.5, 15.6)	0.000
CR plus	62.1 (39.9, 98.6)	9.0 (6.5, 12.9)	0.000	53.6 (30.1, 94.7)	13.9 (8.0, 19.8)	0.000

MPE, malignant pleural effusion; TPE, tuberculous pleural effusion; pfADA, pleural fluid adenosine deaminase; CR, sLDH/pfADA ratio; CR plus, and sLDH/pfADA/pleural fluid L% ratio.

**Table 5:** Diagnostic performance of pfADA, age/pfADA ratio, CR, and CR plus for identifying MPE.

	Cut off value	AUC	Sensitivity	Specificity	PPV	NPV	PLR	NLR	Accuracy
Age ≤50 years:									
pfADA (U/L)	13.0	0.930	88.9%	100.0%	100.0%	98.8%	/	0.11	98.9%
age/pfADA	3.2	0.987	88.9%	100.0%	100.0%	98.8%	/	0.11	98.9%
CR	12.9	0.940	88.9%	88.8%	47.1%	98.6%	7.90	0.13	88.8%
CR plus	25.0	0.930	88.9%	92.5%	57.1%	98.7%	11.85	0.12	92.1%
Age >50 years:									
pfADA (U/L)	9.0	0.844	74.7%	92.3%	95.8%	61.0%	9.71	0.27	80.0%
age/pfADA	6.0	0.855	81.3%	89.7%	94.9%	67.3%	7.93	0.21	83.8%
CR	19.2	0.847	81.3%	87.2%	93.7%	66.7%	6.34	0.21	83.1%
CR plus	22.6	0.836	86.8%	84.6%	92.9%	73.3%	5.64	0.16	86.2%

MPE, malignant pleural effusion; pfADA, pleural fluid adenosine deaminase; CR, sLDH/pfADA ratio; CR plus, sLDH/pfADA/pleural fluid L% ratio; AUC, area under the curve; PPV, positive predictive value; NPV, negative predictive value; PLR, positive likelihood ratio; and NLR, negative likelihood ratio.

**Table 6:** Comparison of AUC among pfADA, age/pfADA ratio, CR, and CR plus.

	Age ≤50 years		Age >50 years	
	Z value	*p* value	Z value	*p* value
pfADA *vs* age/pfADA	-1.436	0.151	-0.208	0.835
pfADA *vs* CR	-0.157	0.875	-0.056	0.955
pfADA *vs* CR plus	0.000	1.000	0.146	0.884
age/pfADA *vs* CR	0.761	0.447	0.174	0.862
age/pfADA *vs* CR plus	0.872	0.383	0.401	0.689
CR *vs* CR plus	0.122	0.903	0.229	0.819

pfADA: pleural fluid adenosine deaminase; CR: sLDH/pfADA ratio; CR plus: sLDH/pfADA/pleural fluid L% ratio; and AUC: area under the curve.
